# Acute Severe Aortic Regurgitation Immediately After Cardiopulmonary Bypass Initiation in a Patient With a Bicuspid Aortic Valve

**DOI:** 10.7759/cureus.116030

**Published:** 2026-09-10

**Authors:** Takumi Yamamoto, Keisuke Sumii

**Affiliations:** 1 Anesthesiology, Saitama Sekishinkai Hospital, Sayama, JPN

**Keywords:** acute severe aortic regurgitation, bicuspid aortic valve, cardiopulmonary bypass, retrograde cardioplegia, transesophageal echocardiography

## Abstract

Antegrade cardioplegia delivery during cardiopulmonary bypass (CPB) is standard practice in cardiac surgery, but it carries a risk of perfusion failure and acute myocardial distension in the presence of aortic regurgitation (AR). In patients with a bicuspid aortic valve (BAV), acute exacerbation of AR can occur immediately after initiating CPB.

An 80-year-old man with a bicuspid aortic valve (Sievers classification type 1: L/R fusion), severe aortic stenosis, mild AR, and an ascending aortic aneurysm underwent elective aortic valve replacement and ascending aortic replacement. Immediately after starting CPB and entering ventricular fibrillation (VF), the patient developed sudden, severe AR (vena contracta: 10 mm). This caused rapid left ventricular (LV) dilation and subsequent dislodgement of the LV vent cannula into the left atrium. Real-time transesophageal echocardiography (TEE) promptly identified both the severe AR and the cannula displacement. Following aortic cross-clamping, antegrade cardioplegia failed due to massive regurgitant volume overload into the LV. Prophylactically placed retrograde cardioplegia via the coronary sinus was immediately initiated, successfully achieving rapid cardiac arrest. The planned surgical procedures were completed successfully without further complications, and the patient was discharged in good condition on postoperative day 11.

In this patient, the acute exacerbation of AR was likely driven by two key factors: first, a rapid elevation in the transvalvular pressure gradient following CPB onset and VF; and second, the unmasking of latent coaptation failure, stemming from BAV asymmetry and calcification, due to the loss of myocardial tone during VF. Real-time TEE monitoring and prophylactic placement of a retrograde cardioplegia cannula are critical for rapid diagnosis, troubleshooting, and ensuring effective myocardial protection.

## Introduction

In Japan, antegrade administration of cardioplegia is routinely practiced during cardiovascular surgery requiring cardiopulmonary bypass (CPB) to ensure sufficient perfusion, uniform distribution, and rapid cardiac arrest [1]. However, this strategy carries a risk of perfusion failure in patients with coronary artery disease, left ventricular hypertrophy, or aortic regurgitation (AR). This is because AR allows cardioplegia to regurgitate into the left ventricle, leading to inadequate myocardial perfusion. Moisa et al. reported that in patients with AR whose intraoperative transesophageal echocardiography (TEE) showed an aortic regurgitant jet width-to-left ventricular outflow tract (LVOT) diameter ratio of ≥0.25, antegrade perfusion alone provided insufficient myocardial protection, necessitating retrograde perfusion [2]. In their cohort, patients requiring retrograde perfusion had a mean jet width-to-LVOT diameter ratio of 0.36 ± 0.06. Furthermore, bicuspid aortic valves (BAV) are prone to sudden AR exacerbation during CPB or ventricular fibrillation, as their inherent structural asymmetry and root abnormalities render leaflet coaptation vulnerable to intraoperative geometric shifts and loss of myocardial tone [3]. Here, we report the successful anesthetic management of a patient undergoing surgery for aortic stenosis (AS) and an ascending aortic aneurysm associated with a BAV. Immediately after starting CPB, the patient developed acute, severe AR. Real-time TEE enabled rapid diagnosis. Although antegrade delivery failed due to the severe AR, a prompt switch to retrograde cardioplegia allowed us to achieve cardiac arrest and complete the procedure safely.

## Case presentation

An 80-year-old man (height: 169 cm, weight: 74 kg) presented to the cardiology department for evaluation of a heart murmur. He was diagnosed with aortic stenosis (AS) accompanied by exertional dyspnea and was referred to our cardiac surgery department for surgical evaluation. On initial examination, his blood pressure was 138/71 mmHg and pulse rate was 69 bpm. Electrocardiography (ECG) revealed a first-degree atrioventricular block and complete right bundle branch block. Preoperative laboratory tests revealed no significant electrolyte disturbances. Preoperative transthoracic echocardiography (TTE) demonstrated a left ventricular ejection fraction of 68%, no regional wall motion abnormalities, a left ventricular end-diastolic diameter of 45 mm, and an end-systolic diameter of 28 mm. The patient was diagnosed with severe AS (peak aortic jet velocity: 4.3 m/s, aortic valve area: 0.74 cm², mean pressure gradient: 40 mmHg), and mild AR. Computed tomography (CT) revealed a bicuspid aortic valve (Sievers classification type 1: L/R fusion [4]) with an aortic valve calcium score of 4,948 measured by the Agatston method, as well as a saccular aneurysm in the ascending aorta (diameter: 46 mm). Coronary CT angiography showed no significant coronary artery stenosis.

The patient was scheduled for aortic valve replacement and ascending aortic replacement under general anesthesia. Anesthesia was induced with midazolam (0.07 mg/kg) and fentanyl (4 μg/kg) and maintained with propofol (2.7 mg/kg/h) and remifentanil (0.1 μg/kg/min). Intraoperative transesophageal echocardiography showed mild aortic regurgitation after induction of anesthesia (Figure 1, Figure 2). To establish CPB, a venous cannula was inserted into the inferior vena cava and an arterial cannula into the ascending aorta. A vent cannula was placed in the left ventricle, and a retrograde coronary perfusion cannula was placed in the coronary sinus (CS) (Figure 3). Five minutes after initiating systemic cooling via CPB, the patient developed ventricular fibrillation (VF), and hypothermia was gradually induced to a core temperature of 27 °C.

**Figure 1:**
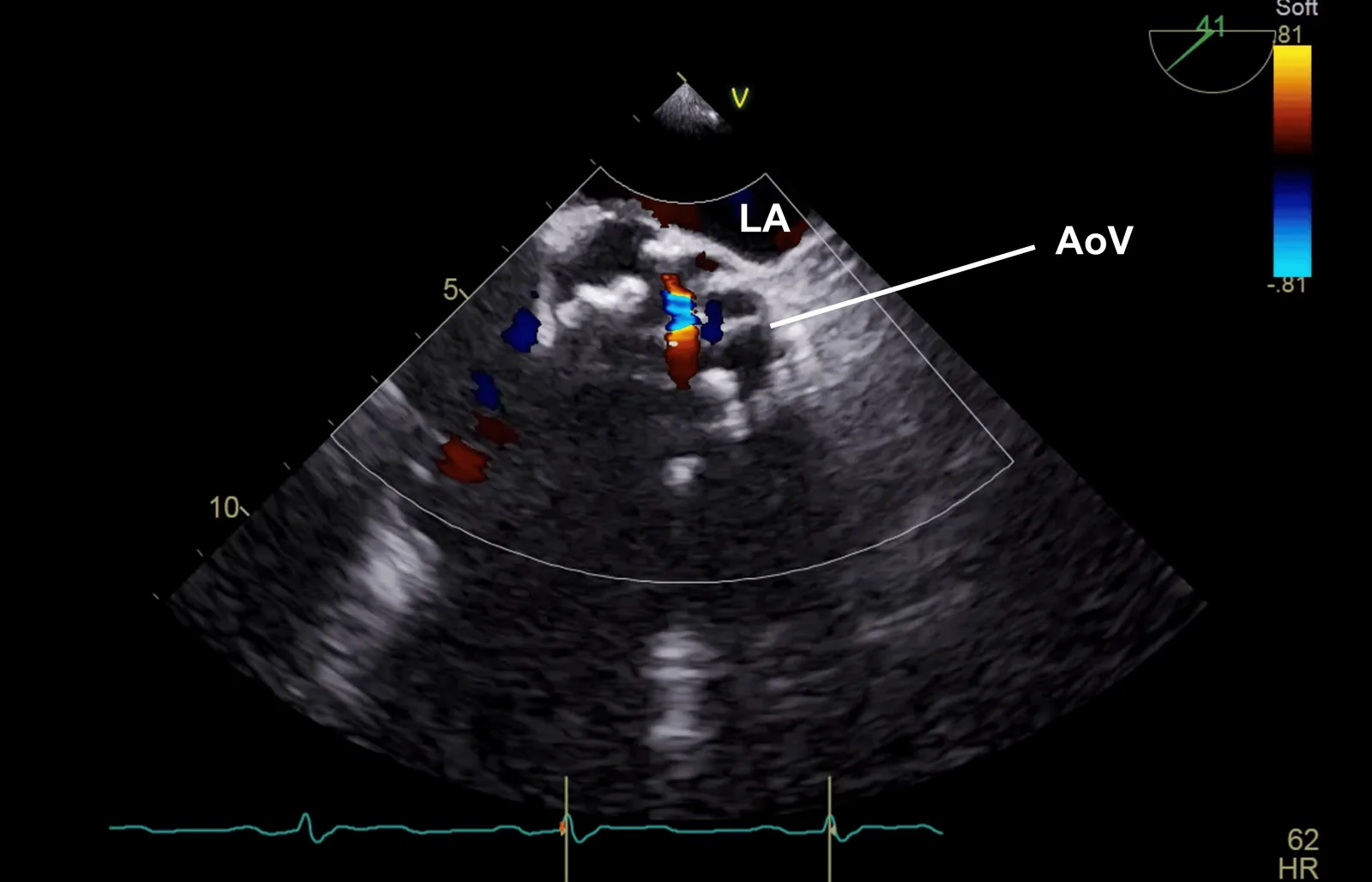
Intraoperative transesophageal echocardiogram after induction of anesthesia Mid-esophageal aortic valve short-axis view demonstrating mild aortic regurgitation following the induction of anesthesia. LA: left atrium; AoV: aortic valve

**Figure 2:**
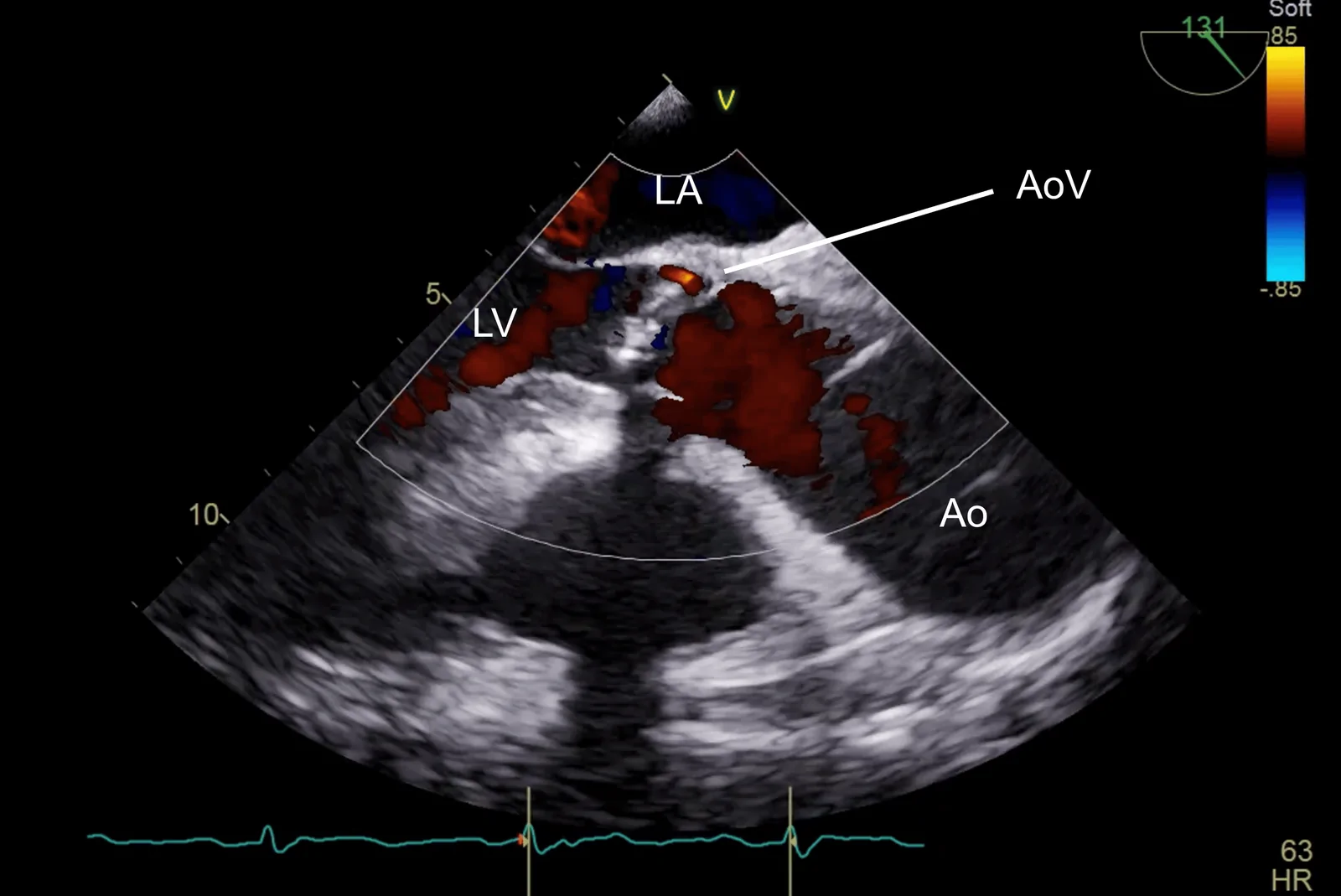
Intraoperative transesophageal echocardiogram after induction of anesthesia Mid-esophageal aortic valve long-axis view demonstrating mild aortic regurgitation following the induction of anesthesia. LA: left atrium; LV: left ventricle; Ao: aorta; AoV: aortic valve

**Figure 3:**
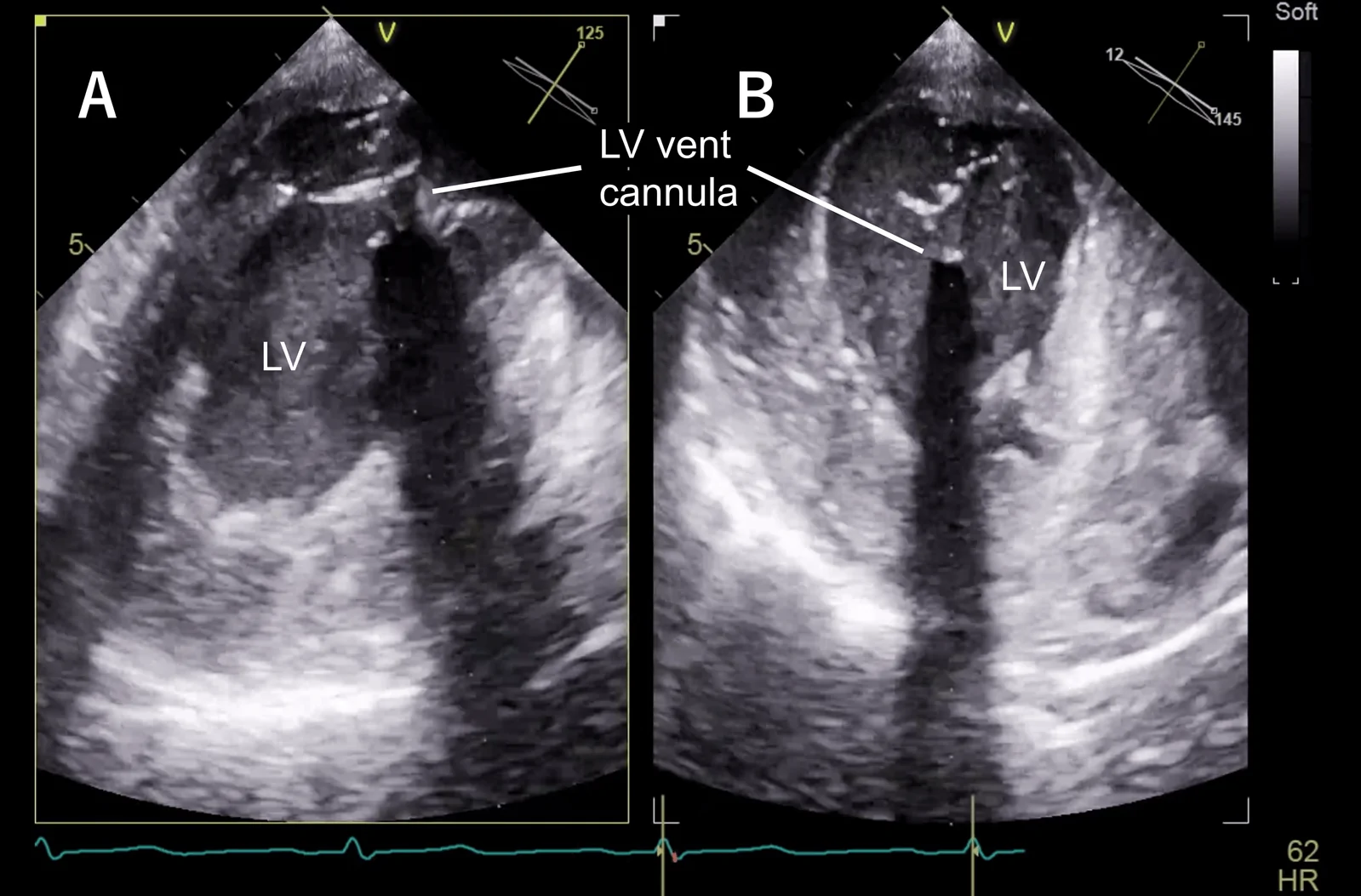
Intraoperative transesophageal echocardiography confirming left ventricular vent cannula placement Transesophageal echocardiography demonstrating the left ventricular vent cannula correctly positioned within the left ventricle (A: transgastric long-axis view, B: transgastric short-axis view). LV: left ventricle

Subsequently, the invasive arterial pressure dropped to 40 mmHg, and TEE revealed acute exacerbation of AR (Figure 4). The AR jet was directed toward the anterior mitral valve leaflet, with a vena contracta (VC) measuring 10 mm. Concurrently, rapid left ventricular dilatation caused the left ventricular vent cannula to become relatively shallow, resulting in its tip dislodging into the left atrium (Figure 5). Immediately following the dislodgement of the vent cannula, the aorta was cross-clamped, and an initial attempt was made to administer antegrade cardioplegia via the aortic root. However, this failed to achieve cardiac arrest because a massive volume of the solution flowed into the left ventricle (Figure 6). We promptly switched to retrograde cardioplegia, which successfully induced immediate cardiac arrest. During retrograde administration, the CS pressure was 15 mmHg, and TEE color Doppler demonstrated a flow velocity of 87 cm/s in the great cardiac vein (GCV) (Figure 7). Subsequently, direct ostial cardioplegia was administered once, after which cardiac arrest was maintained with retrograde perfusion.

**Figure 4:**
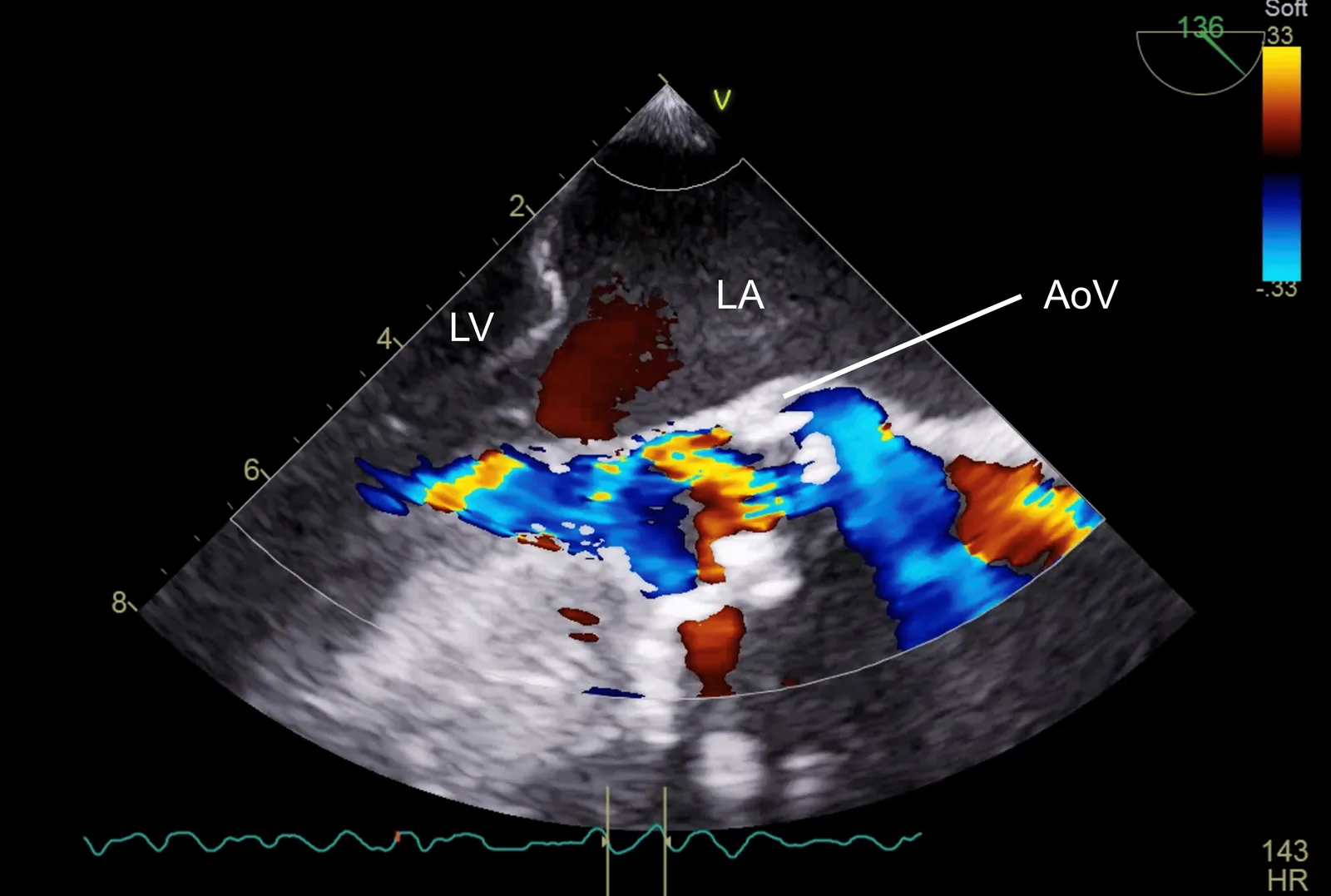
Intraoperative transesophageal echocardiography showing acute exacerbation of aortic regurgitation Transesophageal echocardiography (mid-esophageal aortic valve long-axis view) immediately following the onset of cardiopulmonary bypass, demonstrating acute severe aortic regurgitation with a jet directed toward the anterior mitral valve leaflet and a vena contracta measuring 10 mm. LA: left atrium; LV: left ventricle; AoV: aortic valve

**Figure 5:**
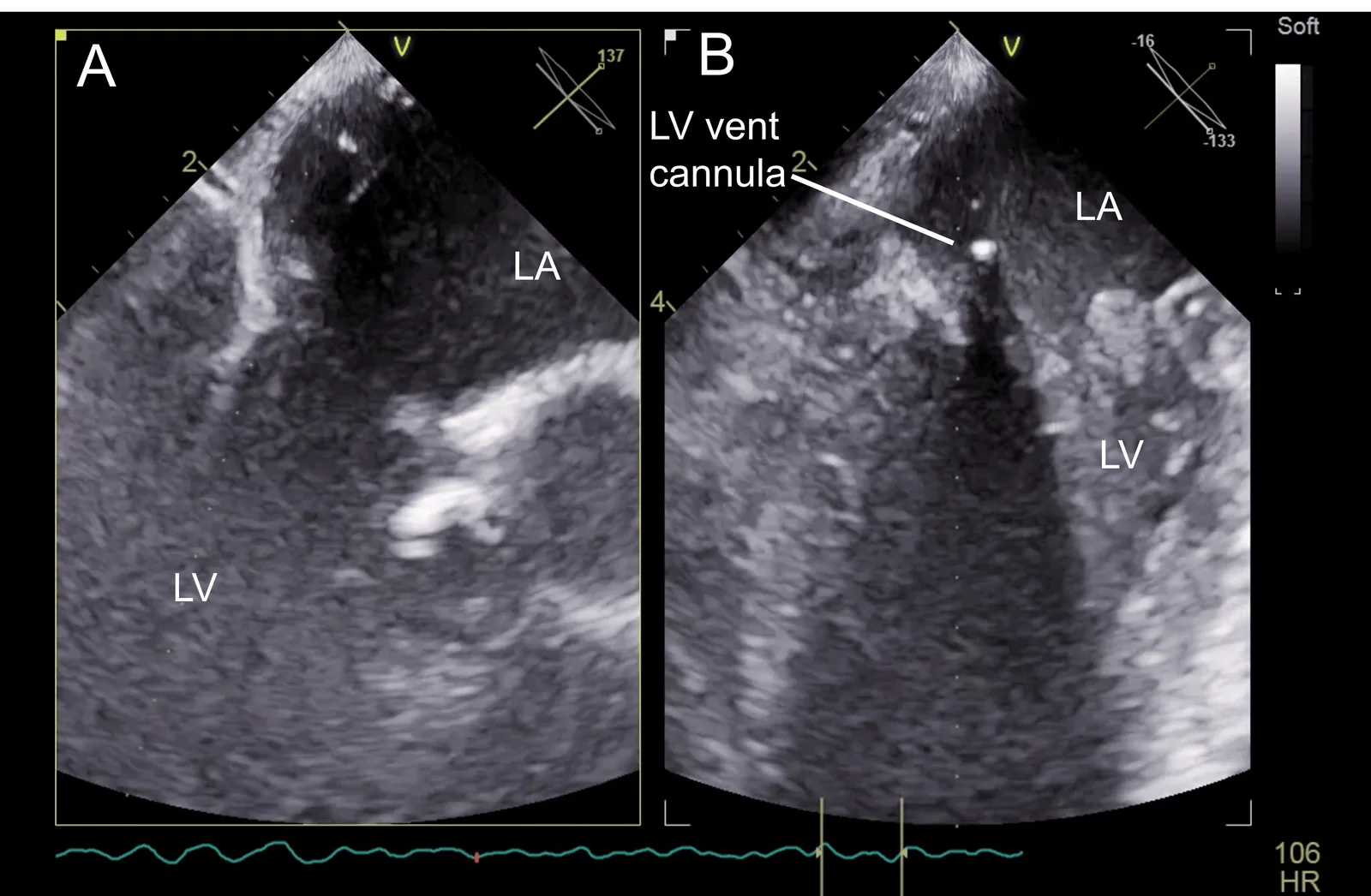
Intraoperative transesophageal echocardiography showing displacement of the left ventricular vent cannula Transesophageal echocardiography showing the tip of the left ventricular vent cannula dislodged into the left atrium following acute left ventricular dilatation and structural alteration(A: mid-esophageal long-axis view, B: mid-esophageal two-chamber view). LA: left atrium; LV: left ventricle

**Figure 6:**
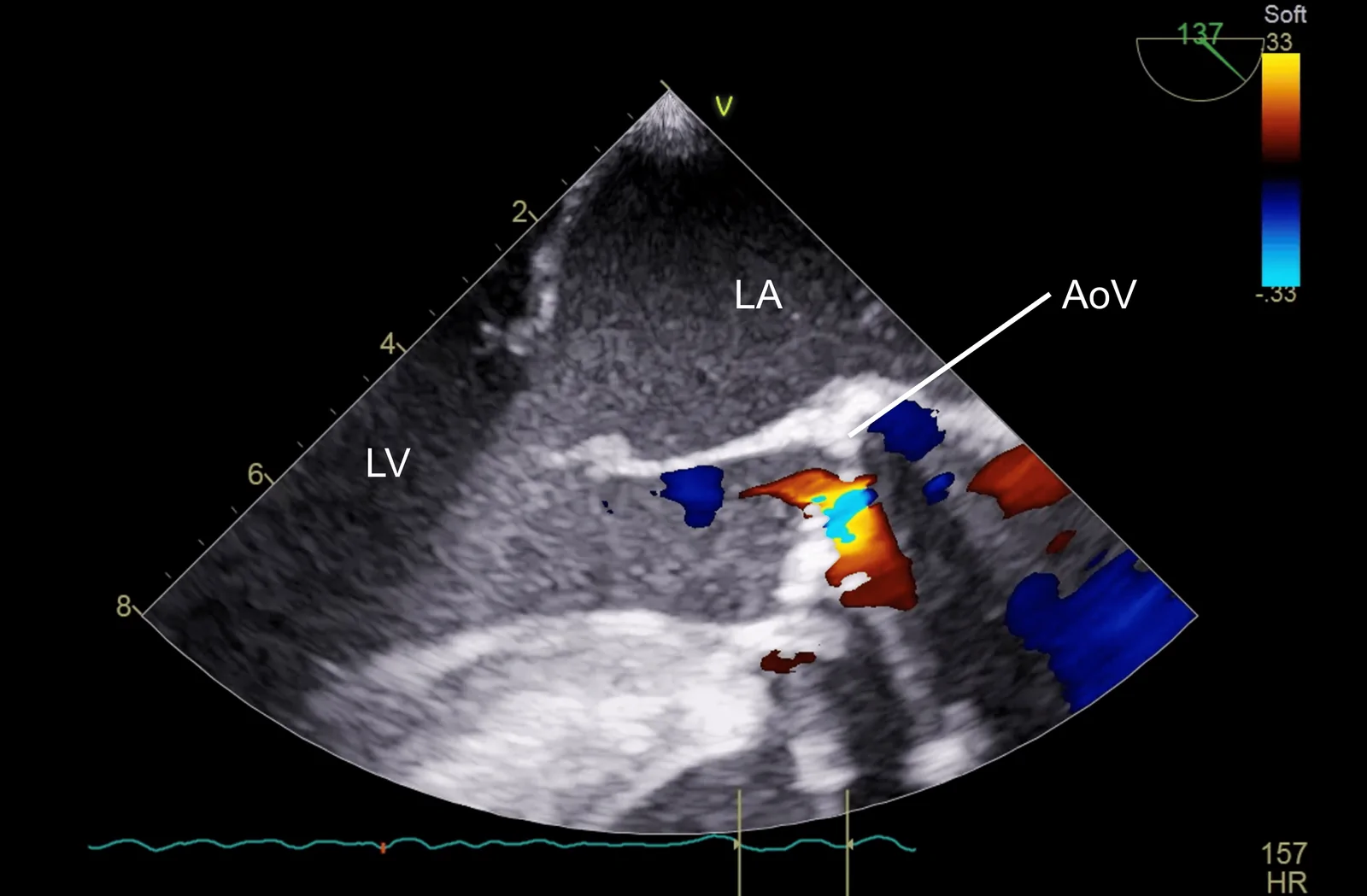
Intraoperative transesophageal echocardiography during antegrade cardioplegia delivery Transesophageal echocardiography (color Doppler) during antegrade cardioplegia administration via the aortic root vent, demonstrating massive inflow of cardioplegic solution into the left ventricle due to severe aortic regurgitation, failing to achieve cardiac arrest. LA: left atrium; LV: left ventricle; AoV: aortic valve

**Figure 7:**
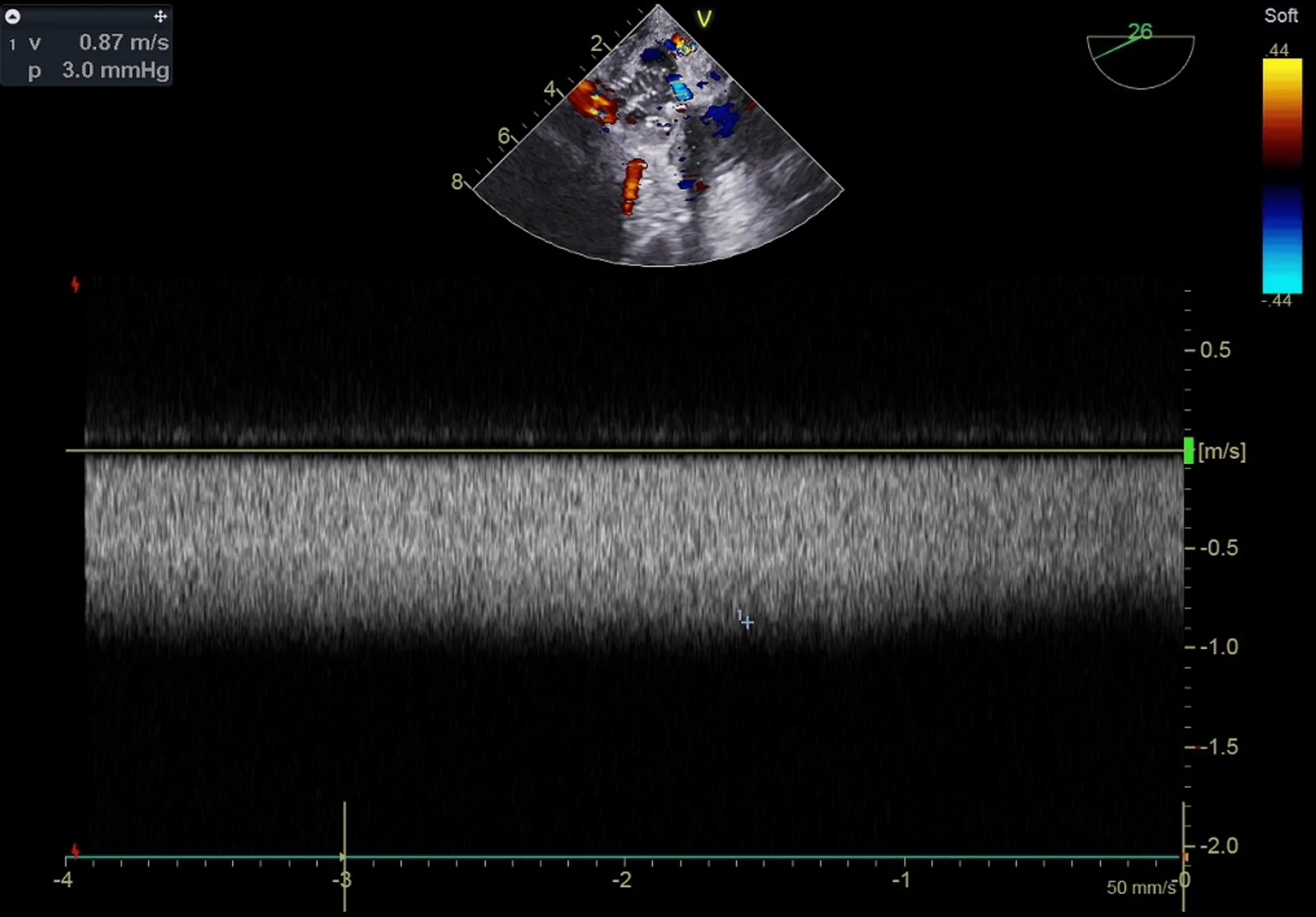
Intraoperative transesophageal echocardiography during retrograde cardioplegia administration Transesophageal echocardiography during retrograde cardioplegia administration, demonstrating adequate retrograde flow in the great cardiac vein with a peak flow velocity of 87 cm/s, leading to prompt cardiac arrest.

Intraoperative inspection confirmed a bicuspid aortic valve (Sievers classification type 1: L/R fusion). Aortic valve replacement and ascending aortic replacement were performed as planned. TEE confirmed no wall motion abnormalities, and the patient was successfully weaned from CPB under inotropic support with norepinephrine (0.09 μg/kg/min) and dobutamine (0.7 μg/kg/min). The postoperative course was uneventful without signs of heart failure or arrhythmia, and he was extubated on postoperative day 1. Follow-up TTE on postoperative day 7 showed preserved wall motion and a well-functioning bioprosthesis without stenosis or regurgitation (peak velocity: 2.1 m/s, effective orifice area index: 1.23 cm²/m²), ruling out patient-prosthesis mismatch (PPM). The patient was discharged ambulatory on postoperative day 11.

## Discussion

The volume of aortic regurgitation is governed by the morphology of the aortic annulus and leaflets, aortic root dimensions, the transvalvular pressure gradient between the aorta and left ventricle, and diastolic duration. In this patient, a marked increase in the transvalvular pressure gradient immediately after CPB onset, along with the post-CPB worsening of leaflet coaptation characteristic of BAV morphology, substantially increased the AR volume.

We estimated the transvalvular diastolic pressure gradient after anesthesia induction based on a combination of directly measured invasive arterial pressure and echocardiographically derived estimations of left ventricular end-diastolic pressure (LVEDP). Preoperative TTE recorded early diastolic mitral annular velocities (e’) of 7.3 cm/s (septal) and 11.2 cm/s (lateral), an early diastolic mitral inflow velocity (E) of 87.0 cm/s, and E/e’ ratios of 11.92 (septal) and 7.77 (lateral), suggesting non-elevated left atrial pressure and LVEDP [5]. Using Nagueh’s formula (pulmonary capillary wedge pressure (PCWP) = 1.24 × E/e’ (lateral) + 1.9) [6], the estimated PCWP (as a surrogate for LVEDP) was 11.5 mmHg. Alternatively, Choi’s formula (LVEDP = 4.9 + 0.62 × E/e’ (septal)) [7] yielded an estimated LVEDP of 12.3 mmHg. Given the directly measured invasive diastolic blood pressure of 45 mmHg at induction, the transvalvular diastolic pressure gradient was indirectly calculated to be 32.7-33.5 mmHg.

Conversely, immediately after CPB onset during VF, transvalvular hemodynamics were evaluated using indirect physiological assumptions rather than direct intracardiac measurements. Left ventricular pressure was not directly measured but was assumed to be approximately 8 mmHg based on previous literature [8]. While the directly measured radial artery pressure was 40 mmHg, central aortic pressure was theoretically estimated at 50-70 mmHg, accounting for the well-recognized peripheral-to-central arterial pressure gradient during CPB (typically 10-30 mmHg higher in the central aorta) [9,10]. Based on these assumed values, the transvalvular pressure gradient during CPB was theoretically estimated as 42-62 mmHg. This acute, presumed elevation in pressure gradient likely exacerbated the AR.

However, a major limitation of this hemodynamic analysis is that the transvalvular pressure gradients were not derived from simultaneous direct measurements of intracardiac (LV) and central aortic pressures, but rather from echocardiographic estimation formulas, peripheral arterial pressure readings, and literature-based physiological assumptions. Furthermore, because intraoperative E and e’ velocities were not obtained immediately post-induction, preoperative TTE values were utilized as surrogates, which may not fully reflect post-induction loading conditions.

In bicuspid aortic valves, acute severe AR can be triggered during CPB. Inherently, BAVs frequently harbor structural asymmetry or aortic root abnormalities, predisposing leaflet coaptation to baseline instability. This delicate mechanism readily fails under intraoperative changes in sinotubular junction (STJ) geometry or reduced myocardial tone [3]. In our patient, in addition to the elevated pressure gradient, marked leaflet asymmetry and severe calcification inherent to the BAV created latent coaptation instability. The transition to VF led to a total loss of forward ejection and a profound drop in myocardial tone, causing this latent coaptation failure to become manifest and resulting in massive AR.

Furthermore, rapid left ventricular distension and structural distortion caused the vent cannula to dislodge into the left atrium. Real-time TEE monitoring allowed us to immediately identify this displacement and rapidly execute troubleshooting steps.

Thus, in patients with a bicuspid aortic valve, even if preoperative AR appears mild, CPB initiation, antegrade cardioplegia delivery, or increased retrograde blood flow can trigger sudden, severe AR due to elevated transvalvular pressure gradients. In our case, pre-placing a retrograde cardioplegia cannula in the CS enabled an immediate and effective transition when antegrade administration failed. Once initial cardiac arrest was achieved via retrograde perfusion, direct ostial cardioplegia was utilized as a definitive route to ensure reliable myocardial protection. Continuous TEE monitoring is crucial for assessing AR severity in real time and verifying cannula positioning to achieve prompt cardiac arrest. Clinicians should maintain high vigilance for this phenomenon in patients with similar anatomical features, as acute AR can also occur in scenarios that increase LV afterload such as veno-arterial extracorporeal membrane oxygenation (VA-ECMO) or high-dose vasopressor administration [11,12].

## Conclusions

Acute, severe aortic regurgitation (AR) can unexpectedly develop immediately after initiating cardiopulmonary bypass (CPB) in patients with a bicuspid aortic valve (BAV), even when preoperative AR is classified as mild. Sudden elevations in the transvalvular pressure gradient combined with loss of myocardial tone during ventricular fibrillation can unmask latent coaptation failure. Real-time transesophageal echocardiography (TEE) is essential for the rapid diagnosis of acute AR and for detecting associated complications such as left ventricular vent dislodgement. Furthermore, prophylactic placement of a retrograde cardioplegia cannula allows an immediate, effective transition to retrograde administration when antegrade perfusion fails. Clinicians should maintain high vigilance for acute AR in BAV patients during CPB or under conditions that increase left ventricular afterload.
